# Differences in brain gene transcription profiles advocate for an important role of cognitive function in upstream migration and water obstacles crossing in European eel

**DOI:** 10.1186/s12864-015-1589-y

**Published:** 2015-05-12

**Authors:** Tomasz Podgorniak, Massimo Milan, Jose Marti Pujolar, Gregory E Maes, Luca Bargelloni, Eric De Oliveira, Fabien Pierron, Francoise Daverat

**Affiliations:** Irstea Bordeaux, UR EABX, HYNES (Irstea – EDF R&D), 50 avenue de Verdun, Cestas, 33612 Cedex France; University of Padova, Viale dell’Università 16, Legnaro, 35020 PD Italy; Department of Bioscience, Aarhus University, Ny Munkegade 114, Aarhus C, DK-8000 Denmark; Centre for Sustainable Tropical Fisheries and Aquaculture, Comparative Genomics Centre, College of Marine and Environmental Sciences, James Cook University, Townsville, Qld 4811 Australia; Laboratory of Biodiversity and Evolutionary Genomics, University of Leuven (KU Leuven), Leuven, B-3000 Belgium; EDF R&D LNHE, HYNES (Irstea-EDF R&D), 6, quai Watier, Bat Q, Chatou, 78400 France; Univ. Bordeaux, EPOC, UMR 5805, Talence, F-33400 France; CNRS, EPOC, UMR 5805, Talence, F-33400 France

**Keywords:** Transcripomics, European eel, Water dams, Microarray, Synaptic plasticity, Fish brain

## Abstract

**Background:**

European eel is a panmictic species, whose decline has been recorded since the last 20 years. Among human-induced environmental factors of decline, the impact of water dams during species migration is questioned. The main issue of this study was to pinpoint phenotypic traits that predisposed glass eels to successful passage by water barriers. The approach of the study was individual-centred and without any *a priori* hypothesis on traits involved in the putative obstacles selective pressure. We analyzed the transcription level of 14,913 genes.

**Results:**

Transcriptome analysis of three tissues (brain, liver and muscle) from individuals sampled on three successive forebays separated by water obstacles indicated different gene transcription profiles in brain between the two upstream forebays. No differences in gene transcription levels were observed in liver and muscle samples among segments. A total of 26 genes were differentially transcribed in brain. These genes encode for, among others, keratins, cytokeratins, calcium binding proteins (S100 family), cofilin, calmodulin, claudin and thy-1 membrane glycoprotein. The functional analysis of these genes highlighted a putative role of cytoskeletal dynamics and synaptic plasticity in fish upstream migration.

**Conclusion:**

Synaptic connections in brain are solicited while eels are climbing the obstacles with poorly designed fishways. Successful passage by such barriers can be related to spatial learning and spatial orientation abilities when fish is out of the water.

**Electronic supplementary material:**

The online version of this article (doi:10.1186/s12864-015-1589-y) contains supplementary material, which is available to authorized users.

## Background

Among anthropogenic environmental alterations, habitat loss and fragmentation are considered as a major threat to biological diversity [1] and dealing with these changes is among the greatest challenges faced by conservation biologists [2]. Habitat fragmentation of aquatic ecosystems is mainly induced by anthropogenic barriers such as dams and weirs [3]. Main effects of human-induced barriers are: (1) modification of abiotic conditions [4], (2) disruption of population and aquatic community structure in subsequent habitats [5-7] as well as (3) disruption of gene flow [8] and (4) biodiversity loss [9].

**Figure 1:**
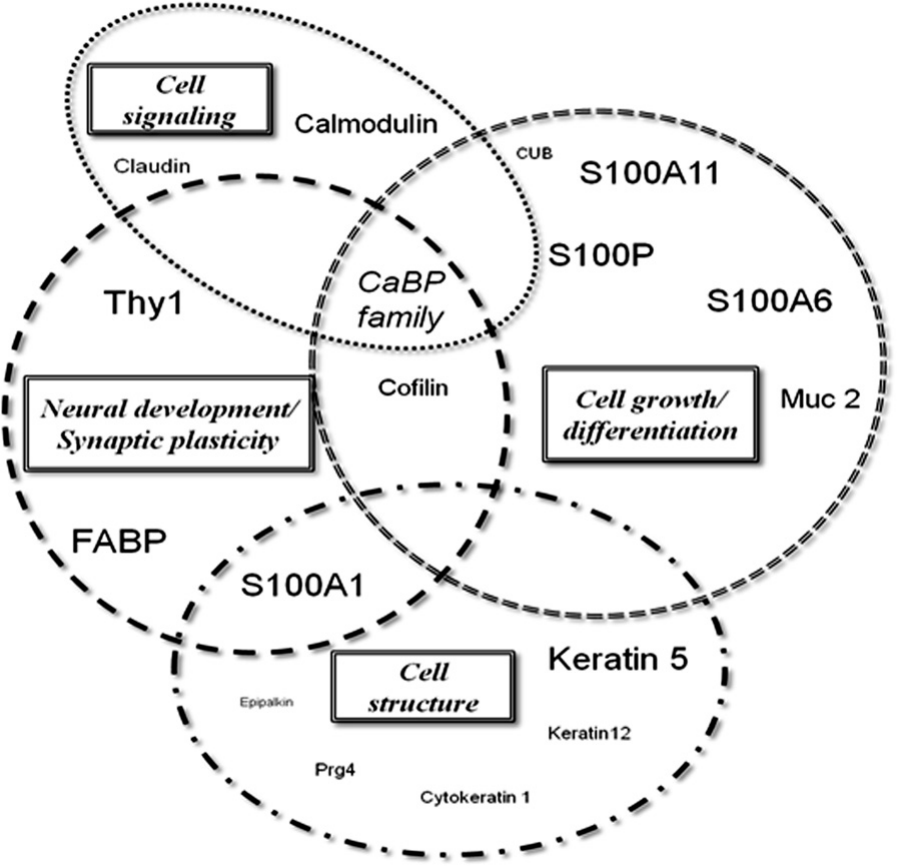
Biological functions of main genes differentially transcribed among segments 3:1 and segments 3:2. The font size is chosen according to the ratios of fold changes; i.e. FC 3:1/ FC 3:2 (Table 4). For more detailed information on each genetic sequence, BLAST statistics, and gene ontology, please see Additional file 1: Table S1.

Endemic species as well as migratory species are the most affected by water impoundment [10,11]. In the case of migratory species, habitat switch can be sometimes imperative in order to reach a particular ontogenetic stage. For diadromous species (i.e. salmonids or eels), growth and reproduction stages require different salinity environments and thus free-flowing corridors between habitats are required to fulfill their life cycle [12]. Therefore, investigating the effects of fragmentation effects on their migratory behavior is of great importance.

European eel *Anguilla anguilla* is a facultative catadromous fish species with a particularly complex life cycle that includes two trans-Atlantic migrations and two metamorphoses. European eel spawns in the remote Sargasso Sea [13]. After spawning, larvae of European eel drift back towards Europe and cross the Atlantic transported by the Gulf Stream system to the coasts of Europe and North Africa. Upon reaching the continental shelf, larvae metamorphose into glass eels and complete the migration into continental (fresh, estuarine and coastal) waters as yellow eels. After a highly variable feeding period in the continent, yellow eels metamorphose into silver eels that migrate back to the Sargasso Sea in a migration of 5,000-6,000 km. Upon reaching the Sargasso Sea, silver adults reproduce once and die. European eel is considered to be critically endangered of extinction with a 90-99% decline observed throughout the distribution range of the species in the last 30 years [14]. The drop in population numbers has affected both recruitment and pre-adult/adult stages and causes of the decline include both anthropogenic (overfishing, man-introduced parasites and diseases, pollution, habitat fragmentation) and natural factors. Among human-related barriers, construction of dams and the consecutive fragmentation of habitats is one of the possible factors contributing to this sharp decline, mainly because water impondment preclude (1) the upstream migration of glass eels to feeding grounds and (2) the spawning migration of adults from the feeding to the spawning grounds in the Sargasso Sea.

Studies addressing the impact of migration barriers on eel upstream migration have mainly focused on quantitative aspects such as mortality of glass eels/elvers downstream of dams due to predation, diseases or intraspecific competition [15]. Such studies also quantified barrier permeability by estimating the abundance of eels on either site thereof. Indeed, many evaluations of single-species specific fishways accounted for passage and attraction efficiency and were only based on the proportion of individuals approaching, engaging and succeeding to pass designed apparatus [16,17]. Although several phenotypic traits are expected to be associated with glass eel upstream migration [18,19], none of them has been studied in the context of passage by water obstacle. In this sense, large individual size [20], swimming speed and high energy reserves could facilitate the success of passage, whereas activity, exploratory behavior and sensibility to environmental cues [21] could increase the probability to find the fishway entrance.

Several authors have proposed a hypothesis of energy- or thyroid- dependent propensity to migrate in glass eels [18,22,23]. According to this hypothesis, the thyroid hormone metabolism is involved in the upstream migration of glass eels. This is in agreement with previous experimental studies that showed that thyroxin (T4) is involved in the migratory behavior of fish [24,25]. A role of the thyroid hormone metabolism in climbing waterfalls has also been suggested in juvenile American eels [26]. Thus, the interindividual variations in thyroid hormone metabolism could be responsible for interindividual variations in “motivation” of juvenile eels to swim against the current and to climb water obstacles.

The aim of the present study was to investigate the interindividual variation of phenotypic traits involved in the passage of water barriers. The chosen approach relied on the comparison of gene transcription (mRNA) patterns among wild glass eels collected below and above successive obstacles dispersed along the same river course.

A non *a priori* approach was chosen to identify individual traits that could differ between downstream and upstream fish. Microarray analysis was used to detect interindividual patterns of gene transcription from a large and functionally diverse set of genes (14,913 annotated contigs [27]) from fish sampled above and directly below the barriers in a river carefully selected for its homogenous conditions.

Three different tissues were sampled: (1) muscle to provide information on fish swimming capacities [28], (2) liver as a proxy for the physiological state of organism [29], and (3) brain, to provide information on perception of environmental cues, arousal, motivation, learning and many other functions involved in behavioral patterns such as those linked to hormone metabolism [22,23].

## Methods

All procedures used in this study were approved by the Aquitaine fish-birds ethic committee (a committee approved and registered by the French Ministry of Higher Education and Research under number 73).

### Sampling site

Canal des Etangs, a freshwater corridor in South-Western France (44.75-44.95 N, 1.1-1.2 W) is a former artificial canal, built in 1850, linking Arcachon Basin to Lacanau Lake. The river line is linear, whereas the water flow remains homogenous and controlled by several weirs. Three successive low-distanced obstacles were built along the river length. The first weir (1,5 m height) is located at 4 km from the tidal limit and equipped with a fish ladder (6 m length, 45° slope) specifically designed for glass eels. It determines the upper limit of the most downstream 4 km section of the canal called segment 1 (Pas du Bouc; +44° 50’ 27.95”, -1° 9’ 8.09”). The second (Langouarde, +44° 51’ 32.92”, -1° 9’ 5.03”) and the third (Joncru; +44° 52’ 57.13”, -1° 8’ 11.70”) weirs are different from the first one, but similar between them; they are larger (2,5 m height) and equipped with identical fishways (rock ramp). The distances from the first weir to the 2^nd^ and 3^rd^ one are respectively 2.3 and 5.3 km (Table 1).

**Table 1:** **Number of pools of tissue samples from each segment used for microarray analyses**

**Sampling site**	**Tissue (number of pools analyzed by DNA microarray analyses)**			**Distance from the 1st segment (km)**
	**Brain**	**Muscle**	**Liver**	
Pas du Bouc (segment 1)	3	3	3	0
Langouarde (segment 2)	3	3	3	2.3
Joncru (segment 3)	3	0	3	5.3

Each pool corresponds to 3 individuals from the same segment. Additionally, the distance from the sampling site to the first segment is shown.

### Sampling

Eels were collected using electrofishing during three consecutive days 6-8^th^ of June 2012 under similar climatic and hydrological conditions. Individuals were sampled below the obstacle, close to the fishway entry in segment 1 and 2. Fish from the segment 3 were sampled on the fishway, as water depth before the obstacle, approximately 2 meters, precluded the use of electrofishing. Ten individuals were selected from each site according to their body size (between 83 and 155 mm) and health status (no externally visible pathogens) to minimize the potential bias. Sampled and selected fish were immediately sacrificed by decapitation and the whole brain (ca. 3.5 mg), liver (ca. 30 mg) and muscle tissues (ca. 40 mg, posterior bottom body part, skin removed) were dissected and stored in separate tubes with RNAlater buffer (1 mL, Qiagen) for gene transcription analysis. Additionally, individual weight was measured for relative condition factor (Kn) calculation [30] and otoliths were extracted for further age analysis.

### Otolith analyses

Sagittal otoliths were embedded in glass slides and submerged by a drop of glue. The otolith was then polished until the core was reached, etched with 10% EDTA, stained with 5% toluidine blue to enhance the annuli and observed with optical microscope (Nikon Eclipse 90i, Japan). For each otolith, age estimation by counting the annuli around the primordium was performed by two independent readers.

### Microarray analyses

Samples of brain, muscle and liver were homogenized by measn of a bead mill homogenizer (45 sec at 3000 oscillations per sec, Mixer Mill MM 200, Retsch). Total RNAs were extracted using the Absolutely RNA RT-PCR Miniprep kit (Agilent) according to the manufacturer’s instructions. A total of 9 fish were used for each sampling site, i.e. 3 pools of 3 individuals by site. RNA quality was evaluated by electrophoresis on a 1% agarose gel. RNA purity and concentration was determined using a NanoDrop spectrophotometry and Agilent 2100 Bioanalyzer. Samples were considered as of good quality RNA when showing A260/280 and A260/230 ratios close to 2 and a minimum RIN (RNA Integrity Number) of 8.

Microarray analysis was conducted using an European eel-specific array consisting of a total of 14,913 probes based on a large collection of high-throughput transcriptomic sequences [27]. Probe sequences and further details on the microarray platform can be found on the GEO database under accession number GPL15124. Sample labelling and hybridization were carried out following the Agilent One-Color Microarray-Based Gene Expression Analysis protocol (Low Input Quick Amp Labelling) [31]. For each individual, 100 ng total RNA were linearly amplified and labelled with the fluorescent dye Cy3-dCTP. In order to monitor microarray analysis work-flow, Agilent Spike-in Mix (a mixture of 10 different viral poly-adenylated RNAs) was added to each RNA sample before amplification and labelling. Labelled cRNA was purified with Qiagen RNAeasy Mini Kit and sample concentration and Cy3 specific activity were measured using a Nanodrop ND-1000 spectrophotometer.

A Cy3 specific activity between 8 and 17 pmol Cy3 per μg cRNA was considered adequate for hybridization. Prior to hybridization, a total of 600 ng of labelled cRNA was fragmented for 30 min at 60°C by adding 5 μl 10X Blocking Agent and 1 μl Fragmentation buffer, and finally diluted with 25 μl 2X GE Hybridization buffer. A volume of 40 μl was dispended into the backing slide, assembled to the microarray slide (each slide containing eight arrays) and placed in the hybridization chamber. Slides were incubated for 17 h at 65°C in an Agilent Hybridization Oven. Afterwards, slides were removed from the hybridization chamber, disassembled in GE Wash Buffer 1, and washed for 1 min in GE Wash Buffer 1 followed by one additional wash for 1 min in GE Wash Buffer 2. Hybridized slides were scanned at 5 μm resolution using an Agilent DNA microarray scanner. Slides were scanned at two different sensitivity levels (XDR Hi 100% and XDR Lo 10%) to increase the power to detect both lowly and highly expressed genes. The two linked images generated were analyzed together. Data were extracted and background subtracted using the standard procedure in Agilent Feature Extraction (FE) software v. 9.5.1. Data was normalized using a quantile normalization procedure using R (http://www.rproject.org/). Normalized fluorescence data from the arrays have been deposited in the GEO database (http://www.ncbi.nlm.nih.gov/geo) under accession number GSE56040. Differentially transcribed genes across samples were identified using the program SAM (Significance Analysis of Microarrays) version 4.0 [32], with the FDR cutoff of 5%. Groups (pools of individuals from each of three segments) were compared using the two-class unpaired test and up-and-down regulated genes were identified. A minimum fold change of 1.5 between groups was considered.

In total, 14,913 genetic sequences were analyzed from brain and liver of 27 individuals (9 pools of three individuals) and from muscle of 18 individuals (no samples from the third segment) (Table 1).

### Quantitative RT-PCR validation of microarray results

A total of 6 genes showing different transcription levels among segments (*claud4, cfl1, s100a1, s100a6, s100a11, thy1*) were chosen to validate the microarray results by means of quantitative real-time Reverse Transcription Polymerase Chanin Reaction (qRT-PCR). For each gene, specific primer pairs were determined using the Primer3Plus software [33] (see Additional file 1: Table S1). Gene transcription level was measured by quantitative real-time Reverse transcribed-Polymerase Chain Reaction (RT-PCR), using the *β-actin* gene as reference. Amplification of cDNA was monitored using the DNA intercaling dye SyberGreen. Real-time PCR reactions were performed in a MX3000P (Stratagene) following the manufacturer’s instructions (one cycle at 95°C for 10 min, and 40 amplification cycles at 95°C for 30 s, 60°C for 30 s and 72°C for 30 s). Each 25 μL reaction contained 1 μL of reverse transcribed product template, 12.5 μL of mix including the SyberGreen fluorescent dye and the enzyme (GoTaq Probe qPCR Master Mix, Promega), 9.5 μL of sterilized pure-water and 2 μL of the gene-specific primer pair at a final concentration of 200 nM for each primer. Reaction specificity was determined for each reaction from the dissociation curve of the PCR product and by electrophoresis. The dissociation curve was obtained by following the SyberGreen fluorescence level during gradual heating of the PCR products from 60 to 95°C. Relative quantification of each gene transcription level was normalized according to the *β-actin* gene transcription. Hence, during our experiment, total RNAs were quantified and a same quantity was used for reverse-transcription. During the subsequent qPCR amplifications, the output cycle corresponding to *β-actin* was examined. This output was always obtained around the same output cycle and no significant variations were observed among conditions, demonstrating the relevance of the β-actin as reference gene in our conditions.

### Statistical analyses

Comparisons among fish groups were performed by analysis of variance (ANOVA), after testing the assumptions of normality (Shapiro-Wilk test) and homoscedascity (Bartlett test) of the error terms. When assumptions were not met, the non-parametric Kruskal Wallis test was used. If significant effects were detected, a Tukey HSD test was used to determine whether means between pairs of samples were significantly different from each other. Computations were performed using R (http://www.r-project.org/).

## Results

### Morphometric data

First, no difference in length or weight were observed among segments (p = 0.548). In addition, no age difference was observed among segments (p = 0.497). At the opposite, the relative body condition was significantly influenced by sampling site (Table 2) and post-hoc analysis indicated significantly lower Kn values in fish sampled in the 3^rd^ segment (Joncru) in comparison with those sampled from the 1^st^ and the 2^nd^ segments (p < 0.001 and p = 0.012 respectively). No difference was observed between the 1^st^ and the 2^nd^ segment (p = 0.12) (Table 2).

**Table 2:** **Size, weight, age and relative condition factor (Kn) of glass eels sampled along the three segments (mean ± SE, n = 9 per site)**

**Origin**	**Length (mm)**	**Weight (g)**	**Age (y)**	**Kn**
Segment 1	125.22 ± 21.59	3.39 ± 1.70	1.33 ± 1.18	1.20 ± 0.13^a^
Segment 2	116.44 ± 19.13	2.32 ± 1.18	0.83 ± 0.72	1.06 ± 0.18^a^
Segment 3	117 ± 14.98	1.81 ± 0.75	1.50 ± 1.05	0.82 ± 0.16^b^

Means designated with different letters (a,b) are significantly different (Tukey’s HSD test, P < 0.05).

### Microarray results

No differences in gene transcription levels were observed in liver and muscle samples among segments. The only differences were observed in the brain tissue (Table 3). Only few differences (n = 5 genes, FDR cutoff = 5%) were observed between segments 1 and 2 (Table 3). A larger number of genes was differentially transcribed when comparing segment 3 with the other two segments: 50 genes between segments 1-3 and 74 genes between segments 2-3 (FDR cutoff = 5%).

**Table 3:** **Number of genes with significant transcription level differences in fish brain sampled along three river segments separated by water obstacles (SAM Pairwise comparison; FC > 1.5; FDR cutoff = 5%)**

**COMPARISON**	**UP-REGULATED**	**DOWN-REGULATED**
Segment 2 vs 1	0	5
Segment 3 vs 1	49	1
Segment 3 vs 2	54	20

For each comparison, the most downstream segment concerned was used as reference.

A total of 40 genes were common to the comparisons between segments 1-3 and segments 2-3. Moreover, all these genes were up-regulated in the most upstream segment. Indeed, these common genes showed a progressive pattern of expression, i.e. the more upstream the segment (or distanced), the more the genes were overexpressed. Differences in regulation of gene expression between the most distanced segments were up to two times higher (shown in bold, Table 4) than those determined between close-distanced segments.

**Table 4:** **Significant fold changes (FC) in gene transcription levels in eels from segment 3 as compared to individuals from segment 2 (FC 3:2) or from segment 1 (FC 3:1) (SAM analysis, FDR cutoff = 5%)**

**NAME**	**FUNCTION**	**EelBase number**	**FC 3:1**	**FC 3:2**	**FC 3:1/FC3:2**
S100P	calcium binding	eeel2_rep_c5535	12.7	6.1	**2.1**
S100P	calcium binding	eeel2_s9035	12.4	6.0	**2.1**
S100P	calcium binding	eeel2_s8956	11.8	6.7	1.8
S100P	calcium binding	eeel2_s8475	9.8	6.1	1.6
S100A11	calcium binding	eeel_rep_c16089	16.1	7.4	**2.2**
S100A11	calcium binding	eeel_rep_c58988	15.1	7.4	**2.0**
S100A11	calcium binding	eeel2_rep_c5969	14.0	7.3	1.9
S100A6	calcium binding	eeel2_s6035	14.3	6.5	**2.2**
S100A1	calcium binding	eeel2_rep_c8719	14.2	7.1	**2.0**
S100A1	calcium binding	eeel_s9222	12.0	9.1	1.3
Keratin 5	cell structure	eeel_rep_c59287	29.2	14.5	**2.0**
Keratin 12	cell structure	eeel2_rep_c5249	5.2	4.4	1.2
Keratin 12	cell structure	eeel_s8804	7.8	8.3	0.9
Keratin 12	cell structure	eeel_c8960	8.0	8.5	0.9
Cytokeratin 1	cell structure	eeel_rep_c58375	6.9	7.2	1.0
Cytokeratin 1	cell structure	eeel_c10204	7.3	7.7	0.9
Keratin	cell structure	eeel_c5504	7.7	8.7	0.9
Keratin	cell structure	eeel_c13622	8.3	9.0	0.9
Non muscle cofilin 1	neuralgrowth	eeel2_s5802	4.8	3.3	1.4
Non muscle cofilin 1	neuralgrowth	eeel2_s5819	4.0	3.0	1.3
Non muscle cofilin 1	neuralgrowth	eeel2_s5889	3.6	3.0	1.2
C59 protein	bacterial infection	eeel_c3624	9.2	3.0	**3.1**
Thy1 protein	surface glycoprotein	eeel_c9925	9.4	3.9	**2.4**
SH3 protein	unknown in brain	eeel2_rep_c5904	10.9	5.0	**2.2**
FABP protein	neural growth	eeel2_s8168	10.3	5.0	**2.1**
Mucin 2	unnown in brain	defence system	13.5	8.4	1.6
Intelectin 1	defence system	eeel2_c833	6.9	4.4	1.6
ATPase, Ca++ transporting	unknown in brain	eeel_2_rep_c8320	12.3	7.8	1.6
Calmodulin	synaptic signaling	eeel_c13874	11.5	7.4	1.6
Claudin 4	tight junction	eeel2_c529	6.1	4.6	1.3
Unnamed	unknown in brain	eeel2_c497	4.6	3.7	1.2
L0C100135339	unknown in brain	eeel2__rep_c4986	12.6	10.3	1.2
C2A protein	unknown in brain	eeel_rep_c35338	2.3	2.0	1.2
Zona pellucida-like protein	unknown in brain	eeel2_s8124	4.6	4.0	1.2
C13antigen	unknown in brain	eeel_c14624	10.2	8.9	1.2
Proteoglycan 4	unknown in brain	eeel2_c3589	2.5	2.3	1.1
Serotriflin	unknown in brain	eeel2_rep_c5326	3.3	3.2	1.1
BRAFLDRAFT_63199	unknown in brain	eeel2_s7960	2.4	2.5	1.0
Epiplakin	unknown in brain	eeel_rep_c28794	4.1	5.9	0.7
Mucin5	unknown in brain	eeel2_c2120	5.3	9.3	0.6

The ratios of fold changes; i.e. FC 3:1/ FC 3:2 equal or superior to 2 are shown in bold. Only sequences with FC ≥ 2 are shown.

### qPCR validation of microarray results

To validate microarray data, the transcriptional level of 6 genes that showed strong variations in their transcription levels among sampling sites was measured by qRT-PCR method. These two independent measures, by microarray and qRT-PCR, of transcript abundance gave consistent results, i.e. similar fold changes were observed (see Additional file 1: Figure S1).

## Discussion

### Energetic costs of obstacle passage

Eels sampled in the upstream and downstream segments of an impounded watercourse did not show any difference in terms of age, weight or length, suggesting that the ontogenetic stage of fish was homogenous along the river. Indeed, size was previously found to be the best proxy to assess the ontogenetic stage of glass eels and its related locomotory behavior [34]. Thus, in the present study, even if an effect of ontogenic stage cannot be completely excluded, it appears unlikely that differences observed in fish brain could be explained by the life stage of fish.

In contrast, the fact that relative body condition (Kn) was lower in eels from the most upstream group in comparison to those sampled downstream could imply that passage of water barriers is an energetically and metabolically requiring event [35]. An alternative hypothesis could be that since glass eels do not feed during their upstream migration [15], the distance covered to reach upstream segments might have reduced their energy reserves [36]. Our results are contradictory to a previous study [37], where tendency to migrate was associated with higher energy reserves. In the present study, no differences in muscle and liver gene transcription among fish groups were found, which suggests that energy was not the main cue explaining the difference in passage behavior. However, it is important to notice that muscle samples from the third segment were missing (Table 3), thus precluding a full inter-segment comparison for this tissue.

### Differences in brain transcriptome profiles associated with upstream migration

Microarray analysis of brain tissue revealed that some genes were overexpressed in fish from the most upstream segment of the river compared to the two downstream sections.

Interestingly, most of these overexpressed genes were common to the comparisons between segments 1-3 and segments 2-3. In addition, the fold changes for most of the genes were found to increase with the number of obstacles crossed by glass eels. The analysis of the biological function of these common genes can provide new insights into the phenotypic traits that are stimulated and/or selected after obstacle passage (Figure 1). Among the 40 common overexpressed genes, 4 genes encoding for proteins belonging to the S-100 family proteins were identified.

The S-100 proteins [38,39] are known to control the intracellular homeostasis of calcium, which is one of messengers mediating the effects of neurotransmitters [40]. Moreover, S-100 proteins are also involved in microtubules and microfilaments synthesis [41,42]. They contribute to a broad spectrum of biological processes in the brain including cell migration, gene expression and neural signaling and activity [43] and even learning and memory at a higher biological level [44]. Calcium signaling is indeed an important pathway controlling neuronal activity, fast axonal flow and memory [45,46]. One of the S-100 members, **S100A6** (Calcyclin), was shown to be highly expressed in rat brain neurons [47], and its suggested functions include cell proliferation, differentiation [48] as well as cytoskeletal rearrangements [49,50] and cellular signal transduction [51]. At a higher biological level, S100A6 was shown to be associated with memory formation in rats [52]. Another S-100 member is **S100A11** (Calgizzarin), involved in regulating growth of cells [53] such as keratinocytes [54]. Interestingly, both S100A11 and S100A6 are specific targets of S100B [55], which is involved in neural plasticity [56]. **S100A1** protein could be associated with synapsin and is involved in calcium dependent synaptic vesicle trafficking [57]. Moreover, an association between exploratory behavior and S100A1 has been suggested in mice [58]. Finally, **S100P** protein is involved in cytoskeletal dynamics [59] and cell proliferation [60].

Another overexpressed gene was the cytoskeletal **Cofilin** (or ADF). This gene encodes for a protein that is involved in actin filament destabilization [61], which in turn allows dendritic development and differentiation, as well as neural polarization in mammalian brain [62] and axonal specialization [63]. Cofilin is also involved in other similar functions, such as axogenogenesis, growth cone guidance and dendritic spine formation [64]. A role in synaptic plasticity in rats [65] and associative learning has been proposed in both rats and mice [66-68].

Another up-regulated gene was **claudin 4**, which is involved in epithelial tight junction [69] which in turn allows intercellular communication. Moreover, epithelial tissue is rich in intermediate filaments and cytokeratins, which could be linked with other genes overexpressed in glass eels found upstream. Thus, the genes encoding for the cytokeratin 1 and keratin 12 were found to be overexpressed in migratory eels. **Keratins** are structural proteins found in neurons and glial cells [70]. Keratins are also known for their transient overexpression during neural differentiation from polymorphic cells in rabbits [71]. **Calmodulin** is a well-studied protein involved in calcium-related [72] synaptic neurotransmission [73] and calcium-dependent gene expression [74]. Together with several transcription factors from the IEG (Immediate Early Genes) family under its regulation, calmodulin is strongly linked to learning and memory [75-78], as found in rats. Indeed, genes belonging to the IEG group are among the first genes regulated in response to environmental stimuli [79]. They are involved in long term potentiating (LTP) and in the establishment of long term memory that requires rapid *de novo* synthesis of proteins [80]. Among the other overexpressed genes, **Thy1** encodes a neural surface glycoprotein that was shown to play a role in axogenogenesis in rats [81] and in olfactory system development in mice [82]. Fatty acid binding protein (**FABP)** are involved in several functions in brain, among which neural development and cognitive processes appear to be common to the functions of other overexpressed genes in this study [83].

None of the overexpressed genes were related to thyroid activity, such as iodothyronine deiodinase type I and III. Thus, the hypothesis of thyroid dependent propensity to migrate [84] or climb obstacles [26] was not supported by our results. In contrast, overexpressed genes in upstream eels were mainly involved in cellular signaling, neural development and differentiation, as well as synaptic plasticity.

### Water obstacle effects on gene expression

The difference in gene transcription in fish brain between the most upstream group and the two others could be interpreted either as a difference in brain development [85] or as a difference in cognitive, learning and memory abilities between groups. Fish brain growth is allometric and development of its various parts is linked to environmental conditions [86-89]. Previous experimental investigations pointed out the association between behavioral flexibility and cognitive abilities [90]. Indeed, personality and coping style concepts were both related to individual capacities in spatial memory and learning abilities, where differential regulation of genes involved in neurogenesis was emphasized [91].

Phenotypic traits highlighted in our study seemed to be related to cognition. In our case study, passage through river impoundments would stand for a hard cognitive task as it involves spatial recognition while climbing the walls and route choice based on perception of visual cues, which is rather unusual for juvenile eels [85]. Indeed, water obstacle passage often requires to climb and to get out of the water, where the extremely developed olfactive system of eel could be less useful than in the aquatic environment, making any behavioral decision demanding higher cognitive appraisal than relying on environmental cues. Passing non-natural obstacles such as water dams could represent a real conundrum for eels and could impede the upstream migration for those with undeveloped or with no ability to develop cognitive functions.

### Gene induction by obstacle crossing?

The overall results suggested a difference in brain functioning between individuals successfully crossing the water obstacles and those situated on the downstream part of water impoundment. Whether gene transcription was temporarily induced by the passage event or whether these differences pre-existed in glass eels before they met the obstacle is difficult to decipher. Indeed, transcriptomic analysis provided phenotypic data, but also represented an intermediary step from genotype towards functional phenotype [92]. Gene expression could be therefore interpreted as a physiological acclimatation or phenotypic plasticity [93,94]. Other studies used gene transcription patterns as indicators of adaptive divergence [95,96].

Several studies on European eel considered trans-generational local adaptation hypothesis as less likely, all the more so because of random mating and absence of habitat choice at least during larval dispersal [97]. Previous studies failed to reveal a clear inter-location genetic heterogeneity of eels across Europe [98], and selection on locally adaptive traits may be too costly for eel [99]. Instead, phenotypic plasticity was hypothesized as the best strategy to deal with habitat heterogeneity in such cases [100,101]. A recent experimental study on American eel has proposed an effect of both origin and environment (salinity), as well as its interaction on gene expression [102]. However, differences in plastic responses were higher between environments within origin than gene expression variation between origins for both rearing environments, suggesting that phenotypic plasticity is not the only cause of phenotypic variation in eel, yet its contribution to the process remains overwhelming in front of (epi) genetic differences related to sampling location.

In our case study, changes in gene transcription profiles could be temporarily induced when eels cross the obstacle. The highest differences in gene transcription in brain were found for fish sampled at the third most upstream group while fish were passing the fishway. The hypothesis of temporary induction of gene transcription while crossing the obstacle could be strengthened, not only by the phenotypic plasticity of eel *per se*, but also by the acknowledged and functionally pertaining high plasticity of the brain [91,103,104].

## Conclusion

Our results showed significant differences in gene transcription in the brain of glass eels sampled above and below the water obstacles. Although the influence of swimming distance on molecular phenotypes has to be taken into account by further analyses of non-impounded watercourse, brain plasticity and cognitive function seem to play an important role in the capacity of glass eels to cross aquatic obstacles. Two main directions for the further studies could be proposed. First, a comparison between climbing and remaining eels within the same location would allow focusing on the climbing event only. Next, the persistence of gene expression patterns could be tested by a long-term common garden experiment, thus explaining its proximate cause by separating the phenotypic plasticity and genetic components.

### Availability of supporting data

Probe sequences and further details on the microarray platform can be found on the GEO database under accession number GPL15124. Normalized fluorescence data have been deposited in the GEO database (http://www.ncbi.nlm.nih.gov/geo/) under accession number GSE56040.

## Additional file

Additional file 1: Figure S1. Comparison of fold changes (FC) between the transcription levels of brain genes encoding for Claudin 4, Cofilin 1, S100A1, S100A11 and S100A6 proteins and Thy-1, obtained by microarray (white bars) and RT-qPCR analysis (black bars). Two fold changes are compared: A) between the segments 3:1 and B) between the segments 3:2. **Table S1.** Sequences of specific primers pairs used in quantitative RT-PCR analyses. **Table S2.** Details about the genes that were differentially transcribed in eels from segment 3 as compared to individuals from segment 2 (FC 3:2) or from segment 1 (FC 3:1).
